# Exploring Interpersonal Justice via the Job Demands–Resources Framework: A Structural Equation Model of Nurse Work Engagement and Burnout in Japan

**DOI:** 10.1155/jonm/8900636

**Published:** 2026-08-29

**Authors:** Nicholas Raposo, Johan Vamstad, Dabin Hwang, Yayoi Saito, Anna Klarare

**Affiliations:** ^1^ Connell School of Nursing, Boston College, Chestnut Hill Massachusetts, USA, bc.edu; ^2^ Center for Civil Society Research, Marie Cederschiöld University, Stockholm, Sweden; ^3^ Graduate School of Human Sciences, University of Osaka, Osaka, Japan; ^4^ Department of Women’s and Children’s Health, CIRCLE, Uppsala University, Uppsala, Sweden, uu.se

**Keywords:** burnout, Japan, job satisfaction, nursing, organizational culture, structural equation modeling, work engagement

## Abstract

**Aim:**

To examine the associations of interpersonal justice with work engagement and burnout among hospital nurses in Japan, and to test whether job satisfaction moderates these relationships.

**Design:**

A cross‐sectional study using structural equation modeling (SEM), grounded in job demands–resources framework.

**Methods:**

Survey data were collected between 2016 and 2017 from 2773 nurses across 10 Japanese hospitals. Interpersonal justice was operationalized using workplace fairness and gender equality indicators. Work engagement and burnout were measured using study‐specific indicators. SEM was used to test direct and moderated pathways, with theoretically justified modifications to the measurement model resulting in acceptable fit (RMSEA = 0.052; CFI = 0.987; TLI = 0.976; SRMR = 0.023).

**Results:**

Interpersonal justice was independently associated with higher work engagement (*β* = 0.28, *p* < 0.001) and lower burnout (*β* = −0.28, *p* < 0.001). Job satisfaction also predicted both outcomes but did not moderate the effects of interpersonal justice. These findings suggest that fair and equal work environments support nurse well‐being regardless of overall job satisfaction.

**Conclusion:**

Interpersonal justice represents an important component of healthy work environments that may inform management interventions aimed at strengthening the nursing workforce in Japan and similar contexts.

**Implications for Nursing Management:**

Nurse managers and healthcare leaders can help promote nurse well‐being by fostering fair and equal workplace interactions through transparent decision‐making, equitable workload allocation and promotion practices, inclusive leadership, and mechanisms for addressing discrimination and unfair treatment. Such efforts may support higher work engagement, lower burnout, and a more sustainable nursing workforce.

## 1. Introduction

The global nursing shortage is expected to worsen throughout the remainder of the decade, with leading organizations projecting a shortage of up to 13 million nurses by 2030 [1, 2]. The current state of the nursing workforce threatens not only workforce sustainability but also healthcare organizational resilience and, ultimately, the quality and safety of patient care [3–5]. Accordingly, this ongoing shortage has been characterized as a global health emergency, underscoring the urgent need for investments in rebuilding a well‐supported nursing workforce [1].

While the nursing shortage is often discussed in terms of workforce numbers, its roots extend beyond recruitment and supply. The sustainability of the nursing workforce is also shaped by nurses’ day‐to‐day experiences of work, particularly the factors that promote engagement and those that contribute to burnout. For example, during the COVID‐19 pandemic, up to 52% experienced burnout [1]. Consequently, work engagement and burnout have become central concepts in efforts to understand and address the global nursing workforce crisis [6].

Work engagement is a positive psychological state characterized by vigor, dedication, and absorption regarding one’s work [7], whereas burnout is a syndrome marked by exhaustion, cynicism, and reduced professional efficacy resulting from chronic occupational stress [8]. Both are consequential in shaping patient, provider, and healthcare system outcomes [9, 10]. Higher nurse work engagement has been associated with improved quality of care, fewer adverse events, greater patient satisfaction, enhanced nurse well‐being, lower turnover, and stronger organizational performance [11, 12]. In contrast, nurse burnout has been linked to poorer quality of care, reduced patient safety and satisfaction, and diminished productivity and organizational commitment [13, 14]. Accordingly, a critical question for nursing and healthcare leaders is which aspects of the work environment may be modified to foster engagement and protect against burnout.

Evidence consistently demonstrates that nurses working in healthy, collaborative, and supportive practice environments report higher work engagement and lower burnout [15]. The job demands–resources (JD‐R) theory [16] provides a comprehensive framework for understanding the relationships between workplace conditions and nurse well‐being. According to JD‐R theory, employee well‐being—including work engagement and burnout—is shaped by the interplay between job demands and job resources. In particular, job demands, such as workload and emotional strain, contribute to burnout, whereas job resources, including support, autonomy, and fairness, promote work engagement. Importantly, certain job characteristics may function as either resources or demands depending on their positive or negative manifestations, as in the example of constructive versus destructive leadership behaviors [17]. This perspective provides a useful lens for understanding how workplace characteristics influence nurses’ well‐being.

Viewed through this framework, the nursing work environment comprises a range of job demands and resources that may influence engagement and burnout [18–22]. Common job demands in nursing include heavy workloads, emotional labor, and role conflicts, whereas key job resources include autonomy, role clarity, and skill utilization [23]. In support of efforts to rebuild the global nursing workforce, considerable attention has been directed toward workplace demands and resources that are potentially modifiable through organizational intervention. One such factor is organizational justice.

Organizational justice refers to employees’ perceptions of fairness in workplace procedures, outcomes, and treatment [24, 25]. In general, organizational justice fosters trust between employees and employers, improves job performance, and promotes job satisfaction [25]. Among nurses, organizational justice has been associated with greater work engagement and reduced burnout, as well as lower exhaustion, depression, and distress, and decreased turnover intentions [20, 26].

A key dimension of organizational justice is interpersonal justice, which reflects the extent to which employees are treated with dignity, respect, and fairness in workplace interactions [24]. At its core, interpersonal justice is grounded in the principle of human dignity, violations of which may include sexism, racism, or other forms of biased, discriminatory, or unfair treatment [27]. Unlike other dimensions of organizational justice, interpersonal justice is enacted not only in formal processes or supervisory relationships but also in routine interactions with coworkers and clients, making it particularly salient in relational work contexts such as nursing [27]. Within the JD‐R framework, interpersonal justice could therefore be conceptualized as a relational work characteristic that functions as a resource when interactions are fair and equal and as a demand when marked by negative experiences such as discrimination or unfair treatment.

Consistent with this conceptualization, researchers have linked interpersonal justice to several important work outcomes. Among healthcare workers in Colombia, higher interpersonal justice was associated with lower burnout [28], and a longitudinal study of Swedish workers found that low interpersonal justice increased turnover [29]. Findings among nurses are more varied. Interpersonal justice has been associated with organizational commitment and turnover intention among Canadian nurses [30] and with job satisfaction among Turkish nurses [31]. However, interpersonal justice was not linked to job satisfaction or organizational commitment among Australian nurses [32], and no significant association with burnout was observed among nurses in the United States [33].

Taken together, the evidence suggests that perceptions of interpersonal justice—like organizational justice more broadly—are shaped by cultural and contextual norms and values, underscoring the need for research across diverse settings [24]. In Japan, prior research among nurses has examined organizational justice at a general level, demonstrating associations with organizational commitment [34], turnover intentions [35], and job satisfaction [21]; however, this body of evidence does not include work engagement or burnout as key outcomes, nor interpersonal justice as a distinct construct. As such, relationships among interpersonal justice, work engagement, and burnout among Japanese nurses remain unexplored.

The purpose of this study was to examine the associations between interpersonal justice and both work engagement and burnout among hospital nurses in Japan. We also examined job satisfaction as a moderating factor, as it is a key correlate of engagement and burnout and has been shown to influence the strength of relationships between workplace factors and employee outcomes [18–22]. Drawing on JD‐R theory, we hypothesized that (1) interpersonal justice would be associated with higher work engagement and lower burnout, and (2) job satisfaction would moderate these associations.

## 2. Methods

This study employed a quantitative, cross‐sectional design using structural equation modeling (SEM). Data were derived from a parent study conducted by Japanese and Swedish researchers evaluating the Japanese model for cooperative healthcare services, details of which have been reported elsewhere [36]. Ethical approval was granted by the Ethics Review Board at the Department of Sociology, Osaka University (No. 2015035), and a data user agreement was approved by the Institutional Review Board at Boston College (No. 25.164.01e). Reporting was guided by the APA Style Journal Article Reporting Standards for Quantitative Research (JARS‐Quant) for studies using SEM.

### 2.1. Setting and Sample

Survey data were collected between 2016 and 2017 from 10 Japanese hospitals [36]. Hospitals were purposively sampled based on size (≥ 300 employees) and location (urban and rural). A census sampling approach was used within each hospital, whereby all employees were invited to participate, including nurses, physicians, administrators, and other healthcare personnel. No inclusion or exclusion criteria were applied. Of the 9510 surveys distributed, 6859 (72%) were returned, with response rates ranging from 37% to 90% across hospitals. The present analysis was restricted to respondents who identified as nurses.

### 2.2. Data Collection

The survey instrument was designed to assess the work environment, work conditions, workplace interactions, workplace social contributions, and employee health in Japanese cooperative and public hospitals [36]. Survey items were drawn from prior literature and related instruments, including the Swedish Work Environment Study [37]. To enhance contextual validity and minimize common method variance, instrument development was informed by prior fieldwork and qualitative data, structured interviews with hospital leadership, and organizational documents such as reports and budgets [36]. A team of Japanese and Swedish researchers subsequently reviewed and adapted the items for the Japanese context, translated them into Japanese, and compiled a machine‐readable paper questionnaire. Pretesting was conducted at three participating hospitals to assess clarity, relevance, and comprehensibility, with no substantial revisions required.

Data collection was coordinated with hospital leaders at each site, who communicated the purpose of the study to employees. Given the potentially sensitive nature of some survey items (e.g., perceptions of colleagues and management), procedures were implemented to ensure anonymity and reduce response bias. An independent survey company oversaw data handling, and questionnaires were distributed and returned via designated hospital contacts. Completed surveys were submitted in sealed envelopes and processed by an external data processing company before being returned to the research team in anonymized form. Survey administration procedures were consistent across hospitals, with the exception that questions regarding family composition and income were omitted at the request of public hospital administrators.

### 2.3. Variables and Measures

#### 2.3.1. Interpersonal Justice

Interpersonal justice, a latent variable, consisted of two observed variables: workplace fairness and gender equality. Workplace fairness was measured by a single item that asked respondents to rate their agreement with the statement, “Everyone is treated fairly at your workplace.” Gender equality was measured by another single item, which asked respondents to rate their agreement with the statement, “You do not experience gender discrimination.” Each was assessed on a five‐point Likert scale from totally disagree to totally agree.

#### 2.3.2. Work Engagement

Work engagement, a latent variable, comprised five observed variables: (1) “Your work is interesting and stimulating,” (2) “Your work is worthwhile,” (3) “You are able to use your skills,” (4) “You are needed in your workplace,” and (5) “Your work contributes to society.” Each was rated on a five‐point Likert scale from totally disagree to totally agree.

#### 2.3.3. Burnout

Burnout, the final latent variable, included four observed variables related to physical and mental health. Two items asked how much physical and mental burden employees derived from their work, rated on a five‐point Likert scale from very little to very much. Two additional items asked how frequently in the past year employees were unable to go to work because of physical or mental stress, rated on a four‐point Likert scale from never to frequently.

#### 2.3.4. Covariates

Based on JD‐R theory, we controlled for the potential confounding effects of personal, family, and work characteristics, as well as job satisfaction. Personal characteristics included age (in years), gender (female or male), and highest educational attainment (high school or less; vocational/technical school or junior college; university). Family characteristics included family composition (single; single with children; couple; couple with children; three‐generation family). Work characteristics included average weekly work hours (≤ 40 h or > 40 h) and duration of current hospital employment (< 5 years; 5–10 years; > 10 years). Overall job satisfaction was rated on a five‐point Likert scale from very dissatisfied to very satisfied.

### 2.4. Data Analysis

#### 2.4.1. Data Screening

All analyses were conducted in Stata 18. Data were thoroughly screened for input errors, outliers, and missingness. Three plausible input errors were identified in the age variable, which were recoded as missing. No other influential or extreme outliers were detected. Descriptive statistics were calculated to summarize sample characteristics and key variable distributions. Assumptions of normality, linearity, and independence were evaluated. Normality was assessed via skewness and kurtosis statistics and visual inspection of histograms for continuous variables. Linearity was checked with two‐way scatterplots, using factor scores for latent variables. Potential nonindependence of observations due to hospital clustering was accounted for in SEM analyses. Variance inflation factors (VIF < 10) and correlation matrices (*r* < 0.8) were generated to examine extreme multicollinearity among observed variables.

#### 2.4.2. Measurement Model Specification, Evaluation, and Modification

**FIGURE 1:**
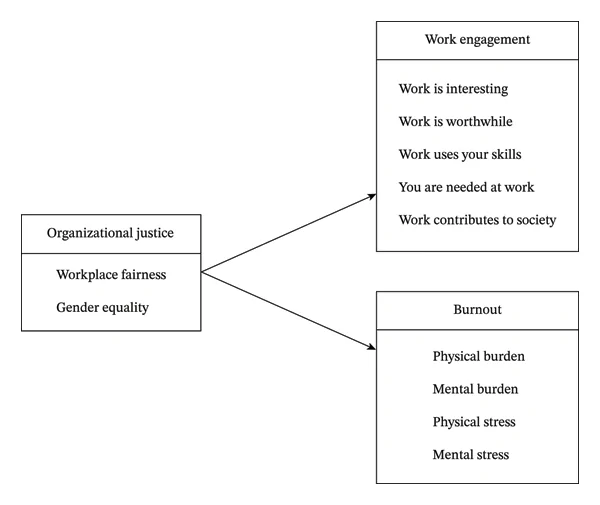
Original measurement model.

We began by mapping our survey variables onto an adapted JD‐R template (Supporting Figure 1). From this, we specified an original measurement model (Figure 1) centered on three key constructs: interpersonal justice, work engagement, and burnout. Based on JD‐R theory and background literature, we conceptualized interpersonal justice as an exogenous variable, and work engagement and burnout as endogenous variables. Confirmatory factor analysis was conducted to evaluate the original measurement model and assess the validity of the latent constructs. Measurement models were estimated using complete‐case analysis.

Model fit was assessed using multiple indices and their recommended thresholds, per Schreiber et al. [38]: standardized chi‐square (*χ* ^2^/df ≤ 2), root mean square error of approximation (RMSEA < 0.06), comparative fit index (CFI ≥ 0.95), Tucker–Lewis index (TLI ≥ 0.95), standardized root mean squared residual (SRMR ≤ 0.08), and Akaike and Bayes information criteria (AIC and BIC, respectively, with lower values indicating better fit). Model modifications were guided primarily by theory, with empirical criteria used to support these decisions. Although a modification index (MI) of 3.84 corresponds to statistical significance at *α* = 0.05 [39], the sensitivity of the *χ* ^2^ statistic to large sample sizes warranted a more conservative threshold. Following established SEM guidelines, MI values greater than 10.0 were used to identify areas of substantial localized strain [40]. Parameters exceeding this threshold were considered for modification only when supported by a strong theoretical rationale, ensuring that adjustments reflected underlying constructs rather than sample‐specific variation.

The measurement model was refined iteratively through this process. For the final measurement model, standardized and unstandardized estimates were generated to facilitate interpretation of factor loadings and structural relationships.

#### 2.4.3. SEM Analysis

After confirming a well‐fitting measurement model, a structural regression model was estimated to examine direct associations between interpersonal justice, work engagement, and burnout, controlling for age, gender, educational attainment, family composition, weekly work hours, duration of employment, and job satisfaction. Clustered standard errors were used to account for potential nonindependence of observations across hospitals. SEM analyses were conducted using Stata’s *mlmv* (full‐information maximum likelihood) estimation, which efficiently handles missing data, yields unbiased parameter estimates, and accounts for clustering [41]. Standardized and unstandardized path coefficients (*β*), along with their significance levels, were reported for all estimated relationships. Standardized coefficients were interpreted as effect sizes using conventional benchmarks [42]. The proportion of variance explained (*R* ^2^) in each endogenous variable was also assessed.

An a priori power analysis was conducted to calculate a minimum sample size for the proposed SEM, which included three latent variables and 11 observed variables. Assuming a small effect size (0.1), desired statistical power of 0.8, and significance level of 0.05, at least 1258 participants were required.

#### 2.4.4. Moderation Analysis

To test whether job satisfaction moderated the effect of interpersonal justice on work engagement or burnout, we generated an interaction term for mean‐centered interpersonal justice and job satisfaction scores (e.g., InterpersonalJustice^∗^JobSatisfaction). This interaction term was added to the SEM with the main effects and covariates. Moderation was determined by the statistical significance of the interaction term [43].

## 3. Results

Personal, family, and work characteristics of the 2773 hospital nurses in the analytic sample are summarized in Table 1. The sample was predominantly female (95%) with an average age of 39 (±11) years. Most nurses had vocational, technical, or junior college education (80%), were partnered with children (45%), worked more than 40 h per week (62%), and had been employed at their current hospitals for fewer than 5 years (40%).

**TABLE 1:** Sample characteristics.

Characteristic	*n*	%
Gender		
Female	2579	95.2
Male	129	4.8
Highest educational attainment		
High school or less	115	4.3
Vocational/technical school or junior college	2155	80.0
University	424	15.7
Family composition		
Single	351	19.0
Single with children	163	8.8
Couple	215	11.6
Couple with children	828	44.8
Three‐generation family	293	15.8
Average weekly work hours		
≤ 40 h	1043	38.1
> 40 h	1696	61.9
Duration of current hospital employment		
< 5 years	1096	39.8
5–10 years	610	22.1
> 10 years	1050	38.1

*Note: N* = 2773. On average, participants were 39.3 (±11.4) years old.

Descriptive statistics for measures of interpersonal justice, work engagement, burnout, and job satisfaction are presented in Table 2. Fewer than half of nurses (45%) somewhat or totally agreed that everyone was treated fairly in their workplace. In contrast, the majority (80%) somewhat or totally agreed that they did not experience gender discrimination at work. While most nurses at least somewhat agreed that their work was interesting and stimulating (53%), worthwhile (66%), and contributed to society (55%), most were neutral about being able to use their skills (40%) or feeling needed in their workplace (40%). Nearly half of nurses (49%) reported rather or very much physical burden of work, and the majority (71%) reported rather or very much mental burden. Despite these challenges, most never missed work due to physical (92%) or mental (91%) stress. Regarding job satisfaction, 38% of nurses were neutral, 35% were rather or very satisfied, and 27% were rather or very dissatisfied.

**TABLE 2:** Distributions of observed variables per latent construct.

Observed variable	*n*	%
Interpersonal justice		
Everyone is treated fairly at your workplace		
Totally disagree	227	8.2
Somewhat disagree	440	15.9
Neutral	857	30.9
Somewhat agree	846	30.5
Totally agree	386	13.9
Missing	17	0.6
You do not experience gender discrimination		
Totally disagree	44	1.6
Somewhat disagree	84	3.0
Neutral	424	15.3
Somewhat agree	1254	45.2
Totally agree	951	34.3
Missing	16	0.6
Work engagement		
Your work is interesting and stimulating		
Totally disagree	151	5.5
Somewhat disagree	348	12.6
Neutral	809	29.2
Somewhat agree	1159	41.8
Totally agree	296	10.7
Missing	10	0.4
Your work is worthwhile		
Totally disagree	98	3.5
Somewhat disagree	269	9.7
Neutral	577	20.8
Somewhat agree	1307	47.1
Totally agree	511	18.4
Missing	11	0.4
You are able to use your skills		
Totally disagree	123	4.4
Somewhat disagree	362	13.1
Neutral	1106	39.9
Somewhat agree	985	35.5
Totally agree	183	6.6
Missing	14	0.5
You are needed in your workplace		
Totally disagree	139	5.0
Somewhat disagree	378	13.6
Neutral	1114	40.2
Somewhat agree	982	35.4
Totally agree	151	5.5
Missing	9	0.3
Your work contributes to society		
Totally disagree	111	4.0
Somewhat disagree	233	8.4
Neutral	895	32.3
Somewhat agree	1228	44.3
Totally agree	296	10.7
Missing	10	0.4
Burnout		
Physical burden of work		
Very little	274	9.9
Rather little	486	17.5
Neutral	642	23.2
Rather much	866	31.2
Very much	491	17.7
Missing	14	0.5
Mental burden of work		
Very little	60	2.2
Rather little	214	7.7
Neutral	538	19.4
Rather much	967	34.9
Very much	981	35.4
Missing	13	0.5
Unable to go to work because of physical stress		
Never	2510	90.5
Rarely	124	4.5
Sometimes	54	2.0
Frequently	39	1.4
Missing	46	1.7
Unable to go to work because of mental stress		
Never	2493	89.9
Rarely	149	5.4
Sometimes	54	2.0
Frequently	34	1.2
Missing	43	1.6
Job satisfaction		
Satisfaction with your work overall		
Very dissatisfied	213	7.7
Rather dissatisfied	521	18.8
Neutral	1047	37.8
Rather satisfied	905	32.6
Very satisfied	69	2.5
Missing	18	0.7

*Note: N* = 2773.

Missingness across observed indicators of latent variables was minimal (< 1.7%). The family composition variable had 33% missingness due to its exclusion from the public hospital surveys, as previously noted; missingness for other covariates ranged from 0.6% to 4.8% (data not shown). Assumptions of normality and linearity were satisfied. Correlations between observed variables (Table 3) were all below 0.8, and the mean VIF was 1.89 (range = 1.12–3.15; data not shown), indicating no concern for extreme multicollinearity.

**TABLE 3:** Correlation matrix.

Observed variable	WF	GE	WE1	WE2	WE3	WE4	WE5	BO1	BO2	BO3	BO4	JS
Workplace fairness (WF)	1.00											
Gender equality (GE)	0.45	1.00										
Work is interesting (WE1)	0.30	0.24	1.00									
Work is worthwhile (WE2)	0.35	0.28	0.77	1.00								
Work uses your skills (WE3)	0.37	0.24	0.61	0.69	1.00							
You are needed at work (WE4)	0.36	0.26	0.47	0.53	0.69	1.00						
Work contributes to society (WE5)	0.26	0.22	0.46	0.54	0.54	0.63	1.00					
Physical burden (BO1)	−0.20	−0.13	−0.16	−0.14	−0.14	−0.18	−0.11	1.00				
Mental burden (BO2)	−0.33	−0.16	−0.25	−0.25	−0.26	−0.24	−0.17	0.43	1.00			
Physical stress (BO3)	−0.06	−0.09	−0.05	−0.06	−0.08	−0.07	−0.06	0.08	0.02	1.00		
Mental stress (BO4)	−0.08	−0.09	−0.05	−0.07	−0.06	−0.07	−0.06	0.05	0.04	0.48	1.00	
Job satisfaction (JS)	0.40	0.21	0.49	0.47	0.48	0.39	0.36	−0.27	−0.41	−0.08	−0.09	1.00

### 3.1. Measurement Model Results

In total, we tested four measurement models; fit indices for each are reported in Table 4.

**TABLE 4:** Measurement model fit indices.

Model	*n*	*χ* ^2^/df	RMSEA	CFI	TLI	SRMR	AIC	BIC
1	2695	48.1	0.132	0.812	0.754	0.109	67,909	68,115
2	2734	51.7	0.136	0.873	0.817	0.107	61,241	61,412
3	2736	26.3	0.096	0.953	0.917	0.105	49,163	49,299
4	2736	8.4	0.052	0.987	0.976	0.023	48,942	49,084

*Note:* *χ* ^2^/df, standardized chi‐squared. Each model is described in‐text.

Abbreviations: AIC, Akaike information criterion; BIC, Bayes information criterion; CFI, comparative fit index; RMSEA, root mean square error of approximation; SRMR, standardized root mean squared residual; TLI, Tucker–Lewis index.

**FIGURE 2:**
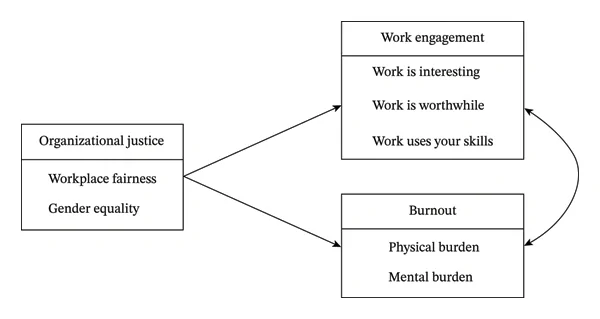
Final measurement model.

Model 1 (Figure 1), representing the original measurement model, produced a poor fit to the data. Model 2 improved fit by excluding physical and mental stresses, which reflected work absences due to stress, as indicators of burnout. This modification was consistent with theoretical definitions of burnout as a subjective experience better represented by perceived physical and mental burden [8]. Refining further, Model 3 excluded two indicators of work engagement (“You are needed” and “Your work contributes to society”) that were more closely aligned with the “meaning of work” [44]. The remaining indicators better represented core aspects of work engagement [7]; model fit improved accordingly. Finally, given that engagement and burnout are understood as related psychological states [16] and have been found to share variances [7], Model 4 allowed the latent constructs to covary. This adjustment resulted in a final measurement model (Figure 2) that demonstrated satisfactory fit. Standardized and unstandardized parameter estimates for this final model are presented in Table 5.

**TABLE 5:** Parameter estimates for final measurement model, structural model, and structural model with moderation.

Effect	Standardized					Unstandardized				
	Estimate	SE^†^	95% CI		*p*	Estimate	SE^†^	95% CI		*p*
			LL	UL				LL	UL	
*Measurement model*										
WF → interpersonal justice	0.82	0.02	0.78	0.87	< 0.001^∗^	1.00				
GE → interpersonal justice	0.54	0.02	0.50	0.58	< 0.001^∗^	0.51	0.03	0.45	0.57	< 0.001^∗^
WE1 → WE	0.83	0.01	0.82	0.85	< 0.001^∗^	1.00				
WE2 → WE	0.92	0.01	0.90	0.93	< 0.001^∗^	1.08	0.02	1.04	1.12	< 0.001^∗^
WE3 → WE	0.75	0.01	0.73	0.77	< 0.001^∗^	0.82	0.02	0.78	0.86	< 0.001^∗^
BO1 → BO	0.51	0.02	0.47	0.56	< 0.001^∗^	1.00				
BO2 → BO	0.84	0.03	0.77	0.90	< 0.001^∗^	1.36	0.11	1.14	1.57	< 0.001^∗^
Error: WF	0.32	0.04	0.25	0.41		0.42	0.05	0.33	0.53	
Error: GE	0.70	0.02	0.66	0.75		0.53	0.02	0.50	0.57	
Error: WE1	0.30	0.01	0.28	0.33		0.32	0.01	0.29	0.34	
Error: WE2	0.16	0.01	0.14	0.18		0.16	0.01	0.14	0.18	
Error: WE3	0.44	0.01	0.41	0.47		0.38	0.01	0.35	0.40	
Error: BO1	0.73	0.02	0.69	0.78		1.11	0.04	1.03	1.19	
Error: BO2	0.30	0.05	0.21	0.43		0.32	0.06	0.22	0.45	
Covariance: interpersonal justice/BO	−0.46	0.03	−0.51	−0.41	< 0.001^∗^	−0.27	0.03	−0.32	−0.22	< 0.001^∗^
Covariance: interpersonal justice/WE	0.49	0.02	0.45	0.54	< 0.001^∗^	0.39	0.02	0.35	0.43	< 0.001^∗^
Covariance: WE/BO	−0.35	0.02	−0.39	−0.30	< 0.001^∗^	−0.19	0.02	−0.22		< 0.001^∗^

*Structural model*										
Interpersonal justice → WE	0.28	0.02	0.24	0.32	< 0.001^∗^	0.52	0.03	0.46	0.59	< 0.001^∗^
Interpersonal justice → BO	−0.28	0.03	−0.34	−0.23	< 0.001^∗^	−0.39	0.06	−0.50	−0.28	< 0.001^∗^
JS → WE	0.44	0.02	0.40	0.47	< 0.001^∗^	0.39	0.02	0.35	0.43	< 0.001^∗^
JS → BO	−0.31	0.04	−0.37	−0.24	< 0.001^∗^	−0.20	0.02	−0.24	−0.15	< 0.001^∗^

*Structural model with moderation*										
Interpersonal justice^∗^JS → WE	0.08	0.10	−0.10	0.27	0.38	0.06	0.07	−0.07	0.19	0.38
Interpersonal justice^∗^JS → BO	0.22	0.15	−0.08	0.52	0.15	0.11	0.08	−0.04	0.26	0.15
Interpersonal justice → WE	0.19	0.08	0.02	0.35	0.03	0.35	0.17	0.03	0.68	0.03
Interpersonal justice → BO	−0.53	0.14	−0.80	−0.25	< 0.001^∗^	−0.72	0.19	−1.08	−0.35	< 0.001^∗^
JS → WE	0.44	0.02	0.41	0.47	< 0.001^∗^	0.40	0.02	0.36	0.43	< 0.001^∗^
JS → BO	−0.29	0.03	−0.34	−0.24	< 0.001^∗^	−0.19	0.02	−0.22	−0.15	< 0.001^∗^

*Note: N* = 2773. Additional abbreviations and full descriptions of each variable are presented in Table 2. Structural models controlled for age, gender, highest educational attainment, family composition, average weekly work hours, and duration of current hospital employment. BO, burnout.

Abbreviations: CI, confidence interval; GE, gender equality; JS, job satisfaction; LL, lower limit; SE, standard error; UL, upper limit; WE, work engagement; WF, workplace fairness.

^†^Robust standard error reported for structural models.

^∗^ *p* < .05.

### 3.2. SEM Results

**FIGURE 3:**
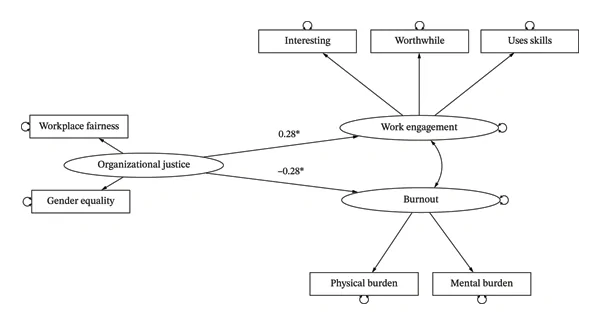
Structural model of organizational justice predicting work engagement and burnout. *Note.* Standardized path coefficients are shown. ^∗^ *p* < 0.05.

The results of the SEM analysis are presented in Table 5, and the model with standardized path loadings is depicted in Figure 3. Controlling for personal, family, and work characteristics, interpersonal justice was positively associated with work engagement (*β* = 0.28; *p* < 0.001; medium effect) and negatively associated with burnout (*β* = −0.28; *p* < 0.001; medium effect). Notably, job satisfaction exhibited stronger associations with both outcomes, showing a medium‐to‐large positive relationship with work engagement (*β* = 0.44; *p* < 0.001) and a medium negative relationship with burnout (*β* = −0.31; *p* < 0.001). Overall, the model explained 38.4% of the variance in work engagement and 36.0% of the variance in burnout.

### 3.3. Moderation Results

**FIGURE 4:**
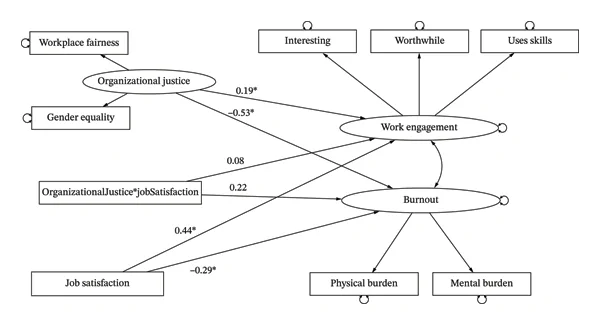
Structural model of organizational justice predicting work engagement and burnout, with moderation by job satisfaction. *Note.* Standardized path coefficients are shown. Moderation by job satisfaction was tested but not supported. ^∗^ *p* < 0.05.

The results of the SEM with moderation analysis are presented in Table 5, and the model with standardized path loadings is shown in Figure 4. The interaction between interpersonal justice and job satisfaction was not significant for either work engagement (*β* = 0.08; *p* = 0.38) or burnout (*β* = 0.22; *p* = 0.15). Nonetheless, the main effects of interpersonal justice and job satisfaction remained statistically significant. Notably, the association between interpersonal justice and work engagement was attenuated (*β* = 0.19; *p* = 0.03; small‐to‐medium effect), whereas its association with burnout was strengthened (*β* = −0.53; *p* < 0.001; large effect), while the effects of job satisfaction were relatively unchanged (work engagement: *β* = 0.44; *p* < 0.001; medium‐to‐large effect; burnout: *β* = −0.29; *p* < 0.001; medium effect). The moderation model explained 38.4% of the variance in work engagement and 35.1% of the variance in burnout.

## 4. Discussion

This study examined associations between interpersonal justice—operationalized as workplace fairness and gender equality—and work engagement and burnout among hospital nurses in Japan and tested whether job satisfaction moderated these relationships. Consistent with our first hypothesis, interpersonal justice was associated with higher work engagement and lower burnout. Contrary to our second hypothesis, job satisfaction did not moderate these associations. The absence of a significant interaction effect alongside significant main effects suggests that interpersonal justice is associated with nurses’ well‐being independently of overall job satisfaction.

Our findings are broadly consistent with prior research in other East Asian contexts, including China and South Korea, where job characteristics have been linked to nurse work engagement [45–47]. They also align with a large study of nurses in China that linked supportive work environments to higher engagement and lower burnout [48]. This study extends prior work by providing evidence from Japan and focusing specifically on interpersonal justice while examining both engagement and burnout, whereas previous research in this context has emphasized broader organizational justice constructs and job attitudes such as satisfaction and commitment.

Although some studies have identified a moderating role for job satisfaction in the relationship between work characteristics and nurse well‐being [49], no such interaction was observed in the present study. Nonetheless, job satisfaction remained a strong predictor of both engagement and burnout, consistent with prior research [22, 50, 51], and altered the magnitude of interpersonal justice effects. Overall, these findings suggest that interpersonal justice and job satisfaction capture related but distinct features of the nursing work environment and may operate in parallel, rather than through a single pathway, to influence nurse well‐being.

Interpreted through the lens of JD‐R theory [16], these findings support the relevance of interpersonal justice as a work characteristic linked to well‐being in the Japanese nursing context. Specifically, our findings suggest that interpersonal justice may encompass both positive and negative manifestations, potentially operating as a job resource when upheld and as a job demand when violated. Although we did not explore underlying mechanisms, based on JD‐R theory, it would follow that workplace interactions perceived as fair and equal may reinforce a sense of value, belonging, and human dignity, thereby supporting work engagement, whereas negative interpersonal experiences, such as unfair treatment or gender discrimination, may function as stressors contributing to burnout.

Within this theoretical framing, the study offers a modest contribution by highlighting the role of interpersonal dynamics—specifically perceptions of fairness and equality—in shaping workplace well‐being, particularly in relational work environments like nursing. While organizational justice has been studied extensively [16], interpersonal justice may represent an important but underexplored component of the resource‐demand dynamic in these settings. Given the study’s limitations, these findings should be interpreted as suggestive rather than conclusive.

## 5. Implications for Nursing Management

The present findings suggest that interpersonal justice may represent a modifiable aspect of the nursing work environment associated with both work engagement and burnout. Accordingly, nursing managers and healthcare leaders should consider interventions that promote fair and equal workplace interactions. Practical strategies include establishing transparent decision‐making, evaluation, and promotion processes, ensuring equitable workload allocation, implementing regular antidiscrimination and antibias training, and fostering inclusive, psychologically safe teams in which nurses feel respected, valued, and able to voice their perspectives and concerns [52, 53]. Supportive leadership practices, meaningful employee involvement in decision‐making, and mechanisms for reporting and responding to unfair or discriminatory treatment may further reinforce perceptions of interpersonal justice within the workplace [54]. Given that interpersonal justice and job satisfaction appear to represent distinct aspects of the work environment, efforts to improve nurse well‐being should address both.

Nurse leaders’ efforts to advance interpersonal justice align with the WHO’s [2] calls to protect nurses’ well‐being and promote gender equality and equity as strategies for strengthening the global nursing workforce. They are also consistent with the WHO’s [55] emphasis on gender‐transformative approaches that address systemic barriers to workforce equity, such as women’s underrepresentation in leadership, persistent pay gaps, and workplace discrimination. Promoting interpersonal justice may, therefore, support not only nurse well‐being but also broader goals related to workforce sustainability, retention, and healthy work environments.

### 5.1. Strengths and Limitations

This study contributes to the literature by examining the role of interpersonal justice, operationalized as workplace fairness and gender equality, in relation to work engagement and burnout among a large, national sample of Japanese nurses using SEM. Nonetheless, the findings should be interpreted in light of several important limitations. Interpersonal justice was measured using study‐specific indicators rather than established multidimensional scales, and work engagement and burnout were assessed using items that do not fully capture their canonical dimensions. Accordingly, the results reflect aspects of perceived workplace conditions and employee well‐being rather than comprehensive representations of these constructs, and the interpretation and practical implications of these findings should be considered accordingly. Additional limitations include the cross‐sectional design, which precludes causal inference; the use of an empirically informed but unvalidated questionnaire; and reliance on single‐item measures, which may limit construct precision. Furthermore, the findings may not be generalizable beyond hospital‐based nurses in Japan and may not fully reflect contemporary nursing workforce conditions, as the data were collected prior to the COVID‐19 pandemic. Future research should employ longitudinal designs and validated, multidimensional measures to more robustly assess these relationships. Examining additional contextual factors, such as leadership practices or team climate, as well as potential moderating and mediating mechanisms, may further clarify how interpersonal justice relates to work engagement and burnout.

## 6. Conclusion

In conclusion, this study provides a context‐specific examination of the relationship between interpersonal justice and nurse well‐being in Japan through the lens of JD‐R theory, demonstrating that perceptions of workplace fairness and equality are associated with both work engagement and burnout. While these findings should be interpreted cautiously given measurement and design limitations, they suggest that interpersonal justice represents a meaningful and potentially modifiable aspect of the nursing work environment. Efforts to promote dignified interpersonal dynamics may help protect nurses’ well‐being while sustaining and retaining valuable human capital, with potential implications for care quality and healthcare system resilience. As such, interpersonal justice may be considered an important component of healthy work environments that may inform management interventions aimed at enhancing nurse well‐being and strengthening the nursing workforce in Japan and similar contexts.

## Author Contributions

All authors contributed to the conception and design of the work. Yayoi Saito, Johan Vamstad, and Anna Klarare acquired data. Dabin Hwang and Nicholas Raposo analyzed and interpreted the data. Nicholas Raposo, Anna Klarare, Dabin Hwang, and Johan Vamstad drafted the manuscript.

## Funding

The parent project was funded by the Japanese Society for the Promotion of Science (JSPS‐15K03913), the Mitsubishi Foundation, and the International Joint Research Program of Osaka University.

## Disclosure

The funders were not involved in the manuscript writing, editing, approval, or decision to publish. All authors read and approved the final manuscript.

## Ethics Statement

The original study protocol was approved by the Ethics Review Board at the Department of Sociology, Osaka University (#2015035), and a data use agreement was granted by the Institutional Review Board at Boston College (#25.164.01e). Research was performed in accordance with the Declaration of Helsinki. Informed consent to participate in the study was obtained from all participants.

## Conflicts of Interest

The authors declare no conflicts of interest.

## Supporting Information

Additional supporting information can be found online in the Supporting Information section.

## Supporting information


**Supporting Information 1** Supporting Figure 1. Adapted job demands–resources (JD‐R) framework. The figure shows the conceptual mapping of study variables onto the JD‐R model, illustrating *inputs* such as personal resources, job resources, and job demands; *mediators* such as work engagement and burnout, leading to *outcomes* for patients, nurses, and healthcare systems.

**Supporting Information 2** This study was reported in accordance with the Journal Article Reporting Standards for Quantitative Research (JARS‐Quant). A completed JARS‐Quant checklist detailing where each reporting criterion is addressed in the manuscript is available as supporting information.

## Data Availability

The data that support the findings of this study are available from the corresponding author upon reasonable request.
