# Supplementary material for: Exploring Interpersonal Justice via the Job Demands–Resources Framework: A Structural Equation Model of Nurse Work Engagement and Burnout in Japan
Source: J Nurs Manag. 2026 Aug 29;2026:8900636. doi: 10.1155/jonm/8900636 (PMC13525545; doi:10.1155/jonm/8900636)
Supplement: Supplementary file 1 [file JONM-2026-8900636-s001.pdf]

JARS–**Quant** | Table 7
Reporting Standards for Studies Using Structural Equation Modeling

Title p. 3

- Mention the basic mechanism or process reflected in the primary model to which the data are fit. (Note: The complexity of the multivariate data analyzed in many structural equation modeling (SEM) studies makes it unlikely that, in most cases, the variables under investigation and the relations between them could be concisely stated in the title.)

Abstract p. 1

- Report values for at least two global fit statistics, each from a different class, and include a brief statement about local fit (residuals). State whether the interpreted model (if any model is retained) is the originally specified model.

Introduction pp. 4-7

- Describe the primary model to be fitted to the data, and include an explanation of theory or results from previous empirical studies that support the primary model.
- Point out paths that are especially important, and justify directionality assumptions, such as the claim that *X* causes *Y* instead of the reverse. Do the same for paths of secondary importance.
- State whether respecification is planned, if the primary model is rejected.

Method pp. 7-12

- State whether the data were collected from research participants or generated by computer simulation.
- Report whether indicators of latent variables were drawn from one questionnaire or from multiple questionnaires.
- Describe, for each questionnaire, whether the indicators are items or total scores across homogeneous sets of items (scales, parcels), stating how
  - scales were constructed, reporting their psychometrics
  - items were treated in the analysis as continuous or categorical
- Report how the target sample size was determined, including
  - rule of thumb
  - availability of resource constraints
  - results of a priori power analysis
  - estimates of parameter precision used to plan the number of cases with appropriate explanation

Method *(continued)*

- For a power analysis, state the
  - target level of power
  - null and alternative hypotheses
  - significance of key parameters
  - fit statistics that figured in the analysis
  - expected population effect sizes
- Report the computer software or algorithm used if the data were generated by simulation, state and justify the sizes of generated samples, and disclose whether samples were lost because of nonconvergence or inadmissible estimates.

Results pp. 14-16

- Report data diagnostics, including
  - percentage of missingness (if some data are missing) and how it is distributed across cases and variables
  - empirical evidence or theoretical arguments about causes of missing data (i.e., missing completely at random [MCAR], missing at random [MAR], or missing not at random [MNAR])
  - evidence that distributional or other assumptions of estimation methods are plausible

Missing Data pp. 10-11

- Indicate the statistical method used to address missingness, such as multiple imputation, full information maximum likelihood (FIML), substitution of values, or deletion of cases. For multiple imputation or FIML estimates, state whether variables not included in the model were specified as auxiliary variables.

Distributions pp. 10, 16-17

- State whether the data were evaluated for estimation methods that assume multivariate normality.
  - Report values of statistics that measure univariate or multivariate skewness and kurtosis that support the assumption of normal distributions.
  - If the data were not multivariate normal, state the strategy used to address nonnormality, such as use of a different estimation method that does not assume normality or use of normalizing transformations of the scores.

© 2024 American Psychological Association

Data Summary pp. 12-17 + Appendices

- Report in the manuscript—or make available in the supplemental materials—sufficient summary statistics that allow secondary analysis, including
  - covariance matrix with means, or correlation matrix with standard deviations and means for continuous variables
  - polychoric correlation matrix, items thresholds, and asymptotic covariance matrix for categorical variables
  - whether the case-level data are archived, and provide information about how those data can be accessed by interested readers.

Specification pp. 10-11, 17-18

- Indicate the general approach that best describes the application of SEM: strictly confirmatory, comparison of alternative models, or model generation.
- Provide the diagram for each model fitted to the data. If the diagram would be overly complex, such as when large numbers of variables are analyzed, then clearly describe the models in text. A reader should be able to translate the text description of a model into a diagram.
- Give a full account of the specification for all models to be evaluated, including observed variables, latent variables, fixed or free parameters, and constrained parameters.
- Report sufficient information, such as tabulations of the numbers of observations versus free parameters, so that the model degrees of freedom can be derived by the reader.
- Verify that models to be analyzed are actually identified. State the basis for this claim, including the method, rules, or heuristics used to establish identification.
- State the basis in theory or results of previous empirical studies if a measurement model is part of a larger model.
- Describe fully the specification of the mean structure if the model has a means component.
- Explain the rationale for including error correlations in the model if correlated error terms are specified.
- Explain how the effects are specified if the model includes interaction effects.
- Explain how nonindependence is accounted for in the model for nested data (e.g., occasions within persons, students within classrooms).
- Describe any comparisons of parameters to be made between groups or occasions, and indicate which parameters are to be compared if models are fitted to data from multiple groups or occasions.

Estimation pp. 10-11

- State the software (including version) used in the analysis. Also state the estimation method used and justify its use (i.e., whether its assumptions are supported by the data).
- Disclose any default criteria in the software, such as the maximum number of iterations or level of tolerance, that were adjusted in order to achieve a converged and admissible solution.
- Report any evidence of an inadmissible solution (e.g., error variances less than zero or constrained by the computer at zero; estimated absolute correlations or proportions of explained variance that exceed 1.0). Explain what was done to deal with the problem.

Model Fit pp. 17-18

- Report fit statistics or indices about global (omnibus) fit interpreted using criteria justified by citation of most recent evidence-based recommendations for all models to be interpreted.
- Report information about local fit, such as covariance, standardized, normalized, or correlation residuals, that justify retaining the model at the level of pairs of observed variables for all interpreted models.
- State the strategy or criteria used to select one model over another if alternative models were compared. Report results of difference tests for comparisons between alternative models.
- State the test and criterion for testing estimates of individual parameters. If parameter estimated were compared over groups or occasions, indicate how those comparisons were made.

Respecification pp. 10-11, 17-18

- Indicate whether one or more interpreted models was a product of respecification. If so, then describe the method used to search for misspecified parameters.
- State which parameters were fixed or freed to produce the interpreted model. Also provide a theoretical or conceptual rationale for parameters that were fixed or freed after specification searching.
- Indicate whether models for which results are presented were specified before or after fitting other models or otherwise examining the data.

Estimates pp. 18-19 + Appendices

- Report both unstandardized and standardized estimates for all estimated parameters.
- Report the corresponding standard errors, especially if outcomes of significance testing for individual parameters are reported. State the cutoffs for levels of statistical significance, if such cutoffs were used.
- Report estimates of indirect effects, both unstandardized and standardized. Also report values of standard errors for indirect effects, if possible. State and justify the strategy for testing indirect effects.
- Report estimates of interaction effects and also results of follow-up analyses that clarify the underlying pattern for interpreted interactions. Also report values of standard errors for such interactions.

Discussion pp. 10-11, 17-18

- Summarize the modifications to the original model and the bases, theoretical or statistical, for doing so.
- Address the issue of equivalent models that fit the same data as well as retained models or alternative-but-nonequivalent models that explain the data nearly as well as retained models. Justify the preference for retained models over equivalent or near-equivalent versions.

© 2024 American Psychological Association
