# Supplementary material for: Exploring Interpersonal Justice via the Job Demands–Resources Framework: A Structural Equation Model of Nurse Work Engagement and Burnout in Japan
Source: J Nurs Manag. 2026 Aug 29;2026:8900636. doi: 10.1155/jonm/8900636 (PMC13525545; doi:10.1155/jonm/8900636)
Supplement: Supplementary file 2 [file JONM-2026-8900636-s002.pdf]

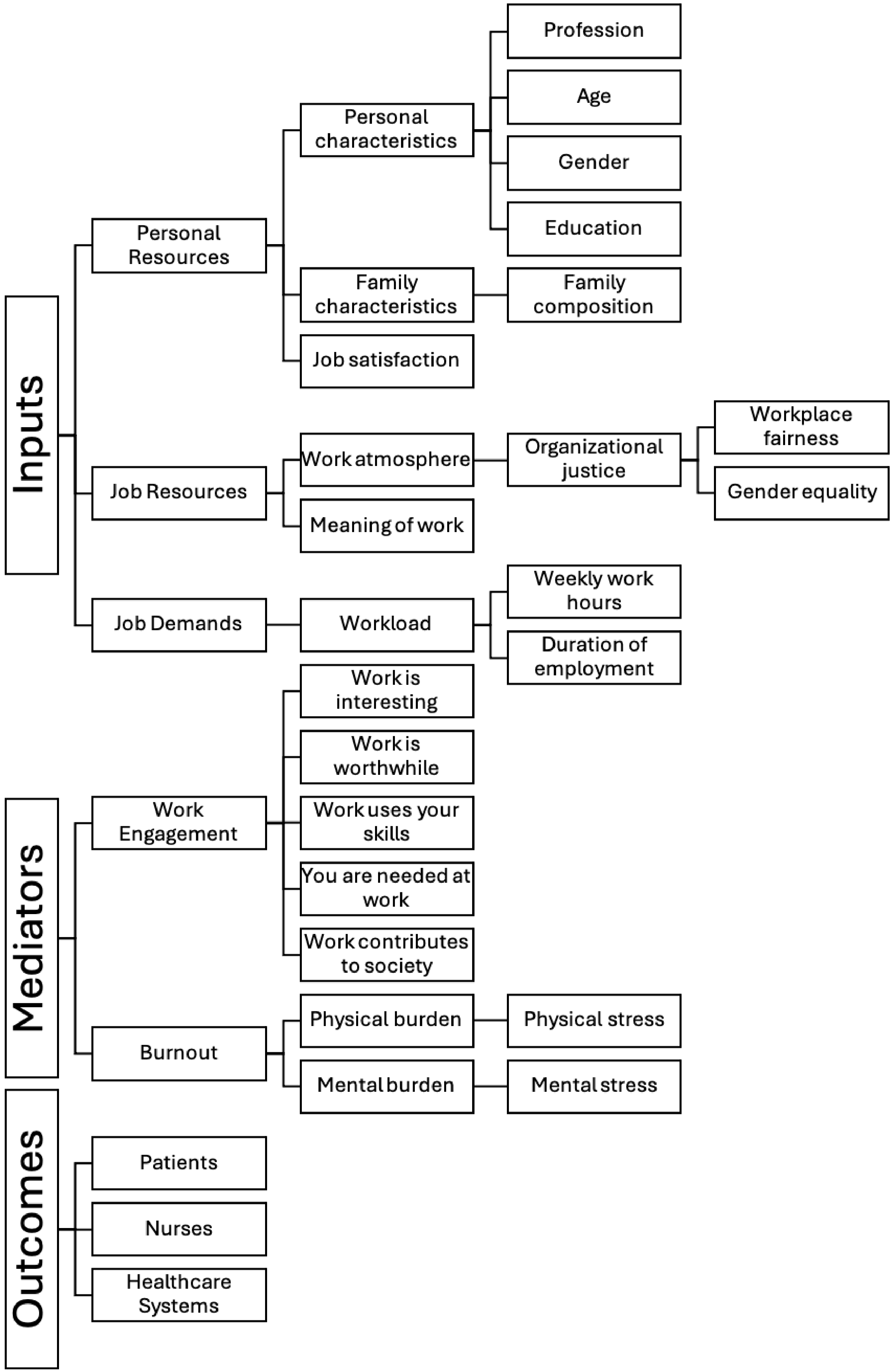
Inputs
Personal Resources
Personal characteristics
Profession
Age
Gender
Education
Family characteristics
Family composition
Job satisfaction
Job Resources
Work atmosphere
Organizational justice
Workplace fairness
Gender equality
Meaning of work
Job Demands
Workload
Weekly work hours
Duration of employment
Mediators
Work Engagement
Work is interesting
Work is worthwhile
Work uses your skills
You are needed at work
Work contributes to society
Burnout
Physical burden
Physical stress
Mental burden
Mental stress
Outcomes
Patients
Nurses
Healthcare Systems
